# Secondary abdominal compartment syndrome required decompression laparotomy during minimally invasive mitral valve repair

**DOI:** 10.1186/s40792-015-0078-5

**Published:** 2016-01-12

**Authors:** Hiroyuki Nishi, Koichi Toda, Shigeru Miyagawa, Yasushi Yoshikawa, Satsuki Fukushima, Daisuke Yoshioka, Tetsuya Saito, Yoshiki Sawa

**Affiliations:** Department of Cardiovascular Surgery, Osaka University Graduate School of Medicine, 2-2, Yamada-Oka, Suita, Osaka 565-0871 Japan

**Keywords:** Minimally invasive surgery, Mitral valve repair, Complication

## Abstract

We treated a 77-year-old patient with secondary abdominal compartment syndrome that caused failure to maintain cardiopulmonary bypass while undergoing elective minimally invasive right mini-thoracotomy mitral valve and tricuspid valve repair procedures. During the operation, a decompression laparotomy was needed to relieve elevated intraabdominal pressure that caused instability of the cardiopulmonary bypass. Due to poor oxygenation and the long cardiopulmonary bypass time, the patient required peripheral extracorporeal membrane oxygenation before recovery. We alert surgeons to this rare complication that can occur even in patients undergoing minimally invasive surgery with a right mini-thoracotomy.

## Background

Secondary abdominal compartment syndrome (ACS) is defined as widespread organ dysfunction in the respiratory, cardiac, renal, and gastrointestinal systems resulting from an increase in intraabdominal pressure that is not associated with a primary abdominal process [1]. ACS frequently occurs after an operation for abdominal trauma or ruptured abdominal aortic aneurysm and is associated with high mortality. This complication may also develop in patients who have not had an abdominal injury or operation, which is referred to as secondary ACS [2]. We report a patient who developed cardiopulmonary bypass instability due to secondary ACS that occurred during minimally invasive mini-thoracotomy mitral and tricuspid valve repair procedures, which was treated with a decompressive laparotomy.

## Case presentation

A 77-year-old man was presented with cardiovascular pathologies that included severe mitral regurgitation (MR) and moderate tricuspid regurgitation (TR). He had no history of congenital venous disease. He had recent shortness of breath with exertion. His New York Heart Association status was class II. Echocardiogram results showed severe MR due to P3 prolapse, moderate TR, and pulmonary hypertension. The left ventricular function was normal with ejection fraction at 61 % and coronary angiogram findings revealed no significant coronary artery stenosis. Renal function was normal with creatinine at 0.69 mg/dl and preoperative hematocrit at 41 %. A preoperative nutritional assessment showed a normal serum albumin level.

The patient underwent a mitral valve repair with P3 resection and suture, followed by ring anuloplasty and tricuspid anuloplasty procedures using a prosthetic ring via a right mini-thoracotomy. Cardiopulmonary bypass was established with antegrade arterial flow through the right subclavian artery, and bicaval venous drainage through the superior vena cava and right femoral vein. We checked the position of venous cannula by transesophageal echocardiography (TEE). Also, we confirmed the position under direct vision when performing tricuspid annuloplasty. Cardiac arrest time was 219 min and cardiopulmonary bypass time until declamping aorta was 262 min. During main procedure, cardiopulmonary bypass flow, mean arterial pressure, and central venous pressure (less than 5 mmHg) were stable within normal range. The value of hematocrit during cardiopulmonary bypass was between 22 and 25 %, and base excess was around −5.0 mEq/L. Blood transfusion was also performed. We observed no sign of poor venous drainage from his lower body, such as severe edema of lower extremity and ascites. While weaning from the bypass, the patient became hemodynamically unstable and the weaning became difficult. Soon thereafter, hemodynamic status worsened even while under the cardiopulmonary bypass due to insufficient venous drainage. TEE showed that the heart appeared to be empty and hyperdynamic contraction of the left ventricle. There was no bleeding in the pericardium cavity or left pleural cavity, and no evidence of aortic dissection. Despite fluid infusion, we could not maintain a sufficient venous return to maintain blood pressure. The only abnormal finding at that point was abdominal distention. We suspected retroperitoneal bleeding but could not find any hematoma throughout the right groin or in transgastric TEE.

**Fig. 1:**
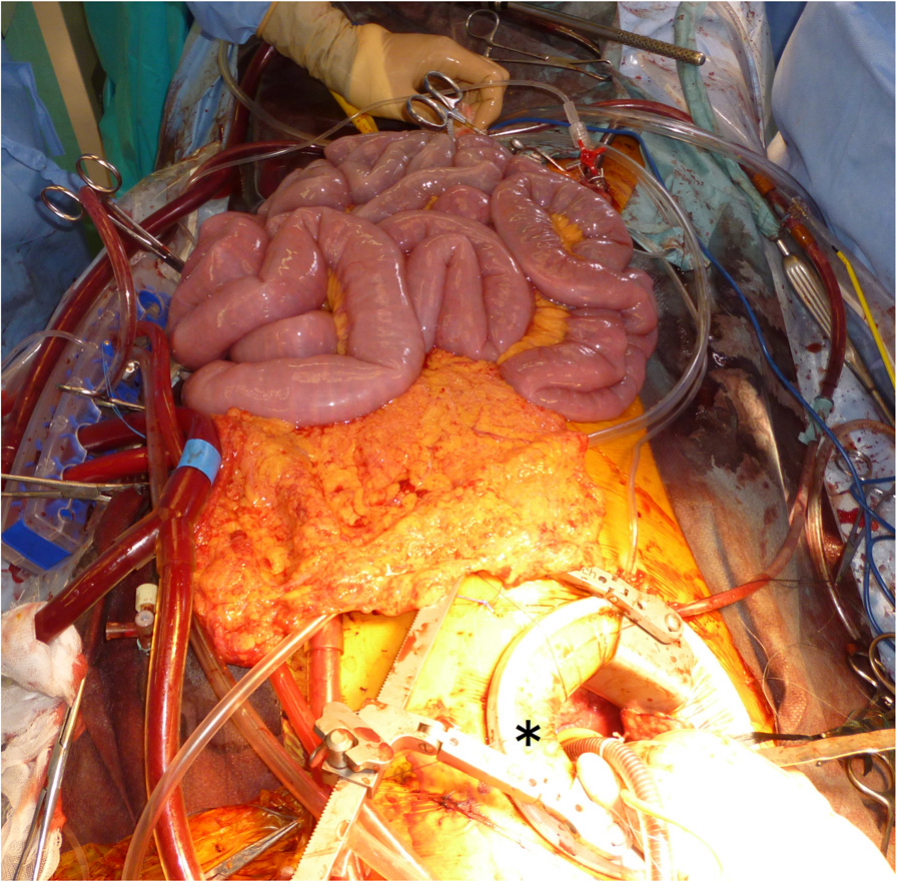
A midline laparotomy revealed severe edema of the small intestine and the mesentery. * right mini-thoracotomy

Since high intraabdominal pressure was externally apparent, we decided to perform a decompression laparotomy, which was done by the general surgery team. A midline laparotomy revealed severe edema of the small intestine and mesentery (Fig. 1). The abdominal cavity was decompressed by putting the small intestine into a transparent plastic intestinal isolation bag, which was secured to the laparotomy wound. As soon as the abdominal cavity was decompressed, central venous pressure dropped down from 15 to 4 mmHg, and the patient became hemodynamically stable under the cardiopulmonary bypass. Because of the long bypass time (501 min) and low oxygenation due to a large amount of fluid infusion, it was difficult to wean off the cardiopulmonary bypass, and the patient was sent to the intensive care unit with extracorporeal membrane oxygenation.

Postoperatively, intestinal and mesenteric edema showed rapid dispersion with high urine output then promptly recovered after the decompression laparotomy. The general surgical team performed abdominal closure on postoperative day 1. Thereafter, the patient became hemodynamically stable and could be weaned from extracorporeal membrane oxygenation on postoperative day 8. Although pneumonia and acute renal failure later developed, he recovered well and was discharged on postoperative day 101. Twelve months after the operation, the condition was stable.

### Discussion

There are two types of ACS; primary with an intraabdominal origin and secondary. Secondary ACS has an extra-abdominal cause such as massive fluid resuscitation leading to intestinal and mesenteric edema [2]. Organ dysfunction seen in these cases typically includes hemodynamic instability, respiratory insufficiency with impaired gas exchange, and acute renal failure, which generally become apparent early in the course of the disease [3]. Therefore, ACS could be fatal in a patient under cardiopulmonary bypass, as increased intraabdominal pressure can cause a failure of venous return. However, only a few cases of ACS during cardiopulmonary bypass have been reported [4, 5], and there are no patients undergoing minimally invasive cardiac surgery.

The mechanism of ACS involves several factors such as shock, hypoxia, massive crystalloid resuscitation, and extreme hemodilution [6]. In addition, there may be some role for extracorporeal circulation in development of intraoperative abdominal hypertension. Previous reports have shown an increase in intraabdominal pressure that is dependent on the degree of hemodilution after initiation of cardiopulmonary bypass, which has a significant impact in such bypass cases with cardiac arrest on mesenteric circulation [7]. Hypothermia is also reported to be a predisposing factor in ACS [4]. Cardiopulmonary bypass is associated with systemic inflammatory response, which can cause increased capillary permeability. Furthermore, insufficient venous drainage frequently results in systemic interstitial edema. In the present case, we initially did not intend to perform transfusion during surgery, though we stored autologous blood obtained prior to the operation, which might have caused hemodilution and a low hematocrit value. Also, when an adequate flow could not be obtained, a large amount of crystalloid infusion was initially given.

Prompt diagnosis is very important to reduce the negative effects of ACS, and once a diagnosis is made, the appropriate treatments should be immediately started. Various nonsurgical approaches, such as intraabdominal fluid drainage and neuromuscular blockade, have been shown to reduce intraabdominal pressure [8]. A decompression laparotomy should be considered to relieve pressure and restore capillary perfusion in the organs of critically ill patients [4, 5]. In the present case, a relatively long period of time was needed to make a diagnosis of ACS, as there were several points to check to determine the reason for instability of cardiopulmonary bypass due to the small incision and insufficient view of the heart, vessels, and other side of the pleural cavity, which might have caused massive fluid resuscitation and requirement of extracorporeal membrane oxygen. Once a decompression laparotomy was performed and intraabdominal pressure relieved, hemodynamic status dramatically improved, and massive urine output led to early abdominal closure.

## Conclusions

This report provides a cautious warning for surgeons by noting that ACS can occur in the setting of minimally invasive cardiac surgery. It is important to be keenly aware of this entity when performing a mini-thoracotomy approach because prompt visual investigation of the entire area is difficult through the small incision. Early recognition and prompt appropriate actions to decrease intraabdominal pressure should be taken to save the life of a patient confronted with this rare complication.

## Consent

Before operation, we obtained a general consent form from the patient for using his clinical data for some clinical studies and registrations. However, the written informed consent for this case report was not obtained from the patient because this report is a retrospective case report without additional invasive treatments for the study.
